# Supplementary material for: Genome-Wide Transcription Start Sites Mapping in Methylorubrum Grown with Dichloromethane and Methanol
Source: Microorganisms. 2022 Jun 27;10(7):1301. doi: 10.3390/microorganisms10071301 (PMC9320726; doi:10.3390/microorganisms10071301)
Supplement: Supplementary file 1 [file microorganisms-10-01301-s001.zip › microorganisms-1775312-supplementary.pdf]

microorganisms

Supplementary Materials

Table S2. Transcription start sites and promoter regions of regulated genes

Gene[1]			Genome position[3]		Promoter region[3]			5′UTR	TSS
**Label**	**Name**	**Product (comment)**			**+1**	**-35**	**-10**	**nt-long**	**class[4]**
**(a) Higher abundance in dichloromethane**									
METDI0008	/	protein of unkown function (only in *Methylorubrum*)	8097	Plus	T	ND	ND	63	P
METDI0091	/	oxidoreductase, 2Fe-2S subunit	91476	Plus	A	TTGCCA	GATATT	18	P
			91470	Plus	G	ND	ND	24	P
METDI0108	/	hypothetical protein of unknown function	110830	Minus	G	ND	ND	48	P
METDI0620	/	conserved protein of unknown function	586227	Plus	A	ND	ND	29	P
METDI1224	/	putative NcrB-like DNA-template transcription regulator of a two-component efflux system	1123413	Minus	A	ND	ND	0	P
METDI1597	/	putative exported protein of unknown function	1464904	Plus	A	ND	ND	17	P
METDI1765	/	protein of unkown function	1634904	Minus	G	TTGACT	TACGCT	25	P
METDI1934	/	putative protein of unknown function within ISMex15 and ISMdi3	1811535	Plus	A	TTGCCG	TAAGAT	102	P
METDI1959	/	periplasmic solute-binding receptor, BugD-like	1833198	Plus	G	ND	ND	217	IP
METDI2115	/	conserved exported protein; putative chemotaxis sensory transducer	1993686	Plus	A	TTCCCG	AAATAT	44	P
METDI2461	/	hypothetical protein of unknown function (only in *Methylorubrum*)	2371778	Minus	A	GTCACT	CATATT	61	P
METDI2655	*dcmR*	transcriptional regulator of DCM dehalogenase	2562332	Minus	C (P_{R1})	TTGCGC	TAACTA	66	P
			2562423	Minus	A (P_{R2})	TTGACG	TTTACT	157	P
METDI2656	*dcmA*	dichloromethane dehalogenase (DCM dehalogenase)	2562877	Plus	G (P_{A})	TTGACA	TATAGA	173	P
METDI2693	/	molybdopterin oxidoreductase	2595011	Plus	T	TTTTTC	TAGGCT	46	P
METDI3043	/	conserved protein of unknown function with 2 CBS domains	2957385	Plus	A	TTGCCA	CACTCG	74	P
			2957334	Plus	T	TTCCGA	CAATAG	125	PAI
METDI3067	/	putative methyl-accepting chemotaxis sensory transducer	2981559	Minus	G	ATTTCG	TAAAAT	43	P
METDI3359	/	protein of unknown function	3291364	Plus	A	TCCACA	TAACTT	0	P
METDI3426	/	conserved protein of unknown function	3347177	Minus	A	GTGACA	TCTTCT	37	P
METDI3569	*arcB*	ornithine cyclodeaminase	3503334	Plus	A	TCGATT	CATGCT	49	P
METDI3571	/	putative acyl carrier protein	3505673	Plus	G	ND	ND	87	IP
			3505666	Plus	G	ND	ND	94	IP
METDI3863	*hppa*	H^+ translocating pyrophosphate synthase	3799402	Minus	G	TGGACG	TAAGTT	104	P
METDI4190	/	protein of unknown function, putative exported portein	4123281	Minus	A	TTTACG	TATCGT	151	P
METDI4584	/	protein of unknown function	4464978	Plus	G	CAGATT	TATACC	50	P

METDI4620	/	putative glutathione-dependent formaldehyde-activating enzyme	4501380	Minus	G	TAGAGC	TAAGAT	95	P
METDI4699	/	transcriptional regulator, AraC family	4585255	Minus	G	TTGCCT	TTAAGT	245	P
METDI4741	/	putative response regulator CheY-like	4637478	Minus	G	ND	ND	87	P
			4637616	Minus	G	ND	ND	225	P
METDI4905	/	putative patatin-like phospholipase	4800535	Minus	G	ND	ND	211	P
METDI4959	/	conserved protein of unknown function	4857811	Plus	G	ND	ND	32	P
METDI5067	/	conserved protein of unknown function	4981146	Plus	A	TTGCCT	TACCTT	87	P
METDI5068	/	putative DNA-binding motif HTH transcriptional regulator	4981626	Plus	G	ND	ND	154	P
			4981632	Plus	A	ND	ND	14<br>8	P
METDI5146	*mxaW*	conserved exported protein of unknown function	5068271	Plus	C	ND	ND	41	P
METDI5190	/	protein of unknown function	5109864	Plus	G	TGGACA	GTTAAT	80	P
METDI5875	/	putative regulator with a cobalamin-binding domain	5868036	Plus	A	TTGCCA	TATCGT	82	P
**(b) Lower abundance in dichloromethane**									
METDI0270	/	protein of unknown function	262250	Plus	A	TTGACG	TGATCT	61	P
METDI0349	/	putative protein disulfide reductase/isomerase, putative thioredoxin	331579	Minus	G	ND	ND	46	P
METDI0384	/	protein of unknown function	357355	Minus	G	ND	ND	82	P
METDI1416	/	transposase of the insertion element ISMdi10, IS110 family	1286085	Minus	A	ND	ND	229	P
METDI1753	*eshA*	cyclic nucleotide-binding protein	1623245	Plus	G	TTGACC	TAGTAC	210	P
METDI1754	/	putative cysteine desulfurase (SufS domain)	1624775	Plus	G	ND	ND	58	IP
METDI1761	/	conserved exported protein of unknown function	1631616	Plus	G	ND	ND	27	P
METDI1982	/	putative ABC transporter substrate-binding protein	1857504	Minus	A	ND	ND	29	P
METDI1983	/	cytochrome c550	1857618	Plus	A	ND	ND	19	P
METDI2867	*glnK*	nitrogen regulatory protein P-II	2797153	Plus	G	TTTATG	TTATTT	188	P
METDI2873	*fdh4B*	formate dehydrogenase subunit B	2808782	Minus	A	ND	ND	114	IP
METDI2874	*fdh4A*	formate dehydrogenase subunit A	2811072	Minus	A	ND	ND	92	P
METDI3157	*glnII*	glutamine synthetase, type II	3075246	Minus	G	ND	ND	192	P
METDI4552	/	protein of unknown function	4439197	Plus	A	ND	ND	68	P
METDI4565	/	conserved protein of unknown function, UspA-like domain	4451683	Minus	G	TCGGCA	TAGGAT	221	IP
METDI5284	/	protein of unknown function	5218043	Minus	A	ND	ND	35	P

[1]Genes with higher or lower mRNA abundance in cells cultivated with dichloromethane or methanol as sole carbon and energy source [12, 49]; [2]Nucleotide position on *M. extorquens* DM4 genome (accession n° FP103042.2); [3]PromoterHunter prediction [50]. ND, when not predicted; [4]TSSAR transcriptional start site categorization: P for primary, I for internal, A for antisense [33].

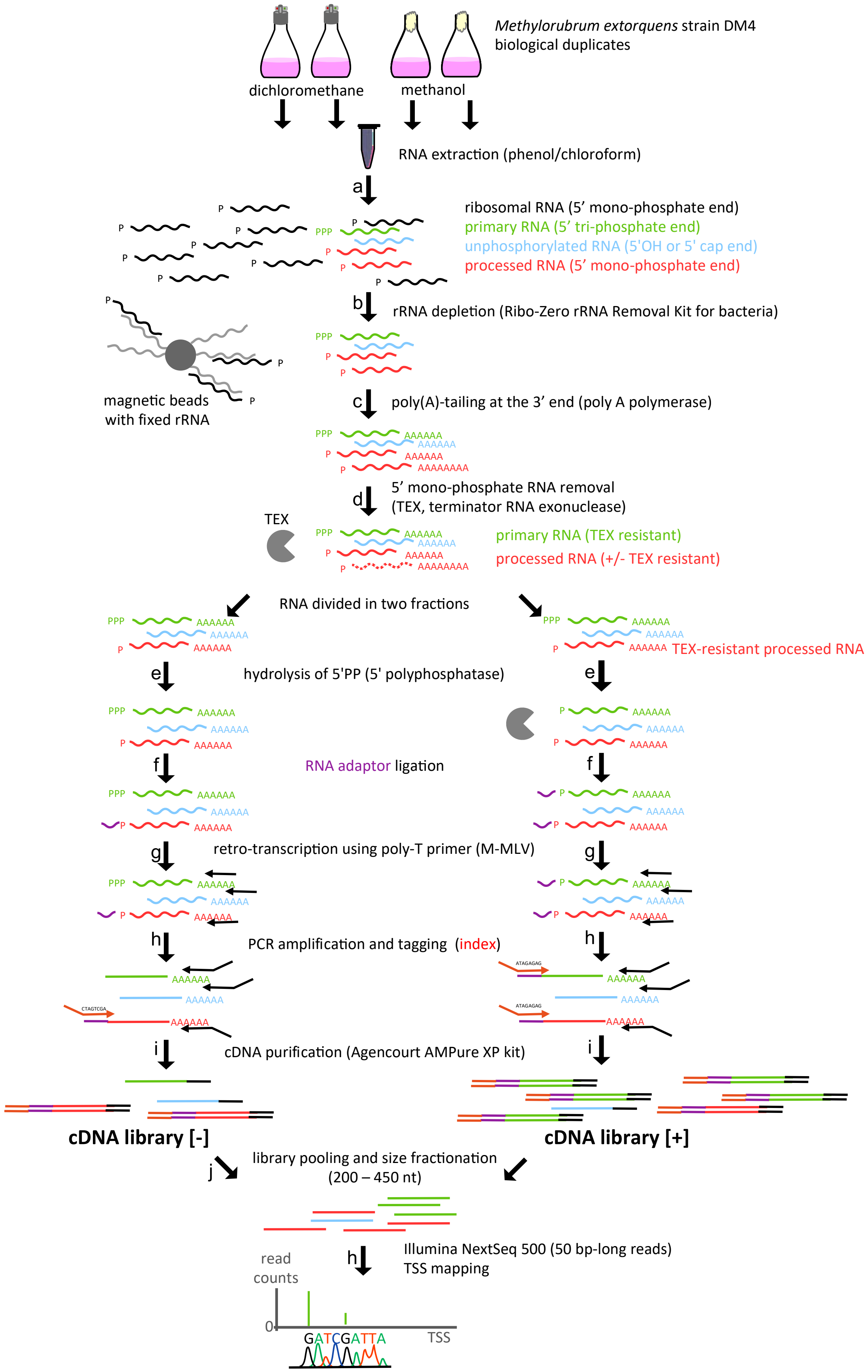
Methylorubrum extorquens strain DM4
biological duplicates
dichloromethane
methanol
RNA extraction (phenol/chloroform)
a
ribosomal RNA (5’ mono-phosphate end)
primary RNA (5’ tri-phosphate end)
unphosphorylated RNA (5'OH or 5' cap end)
processed RNA (5’ mono-phosphate end)
b
rRNA depletion (Ribo-Zero rRNA Removal Kit for bacteria)
magnetic beads
with fixed rRNA
c
poly(A)-tailing at the 3’ end (poly A polymerase)
d
5’ mono-phosphate RNA removal
(TEX, terminator RNA exonuclease)
TEX
primary RNA (TEX resistant)
processed RNA (+/- TEX resistant)
RNA divided in two fractions
TEX-resistant processed RNA
e
hydrolysis of 5'PP (5' polyphosphatase)
f
RNA adaptor ligation
g
retro-transcription using poly-T primer (M-MLV)
h
PCR amplification and tagging (index)
i
cDNA purification (Agencourt AMPure XP kit)
cDNA library [-]
cDNA library [+]
j
library pooling and size fractionation
(200 – 450 nt)
h
Illumina NextSeq 500 (50 bp-long reads)
TSS mapping
read
counts
0
GATCGATTA
TSS

Figure S1. Construction of 5′-end-mapping libraries and cDNA sequencing. rRNA-depleted DNA-free RNAs were obtained from *M. extorquens* aerobically-grown mid-phase cultures, as previously described [12]. After poly(A)-tailing using poly(A) polymerase (**c**), all samples were treated with the processive 5'-to-3' RNA exonuclease (TEX for terminator exonuclease) that digests RNA having a 5'-monophosphate (processed RNAs) (**d**). TEX-resistant RNAs include those with a 5-triphosphate such as that found on the 5'-end of primary bacterial RNA transcripts, those with a 5'-hydroxyl group, and double-stranded RNAs. In this protocol, all RNAs were TEX-treated for primary RNA enrichment and depletion of processed RNA including of 16S and 23S rRNA that escaped step (**b**). Samples were split in two fractions for library (-) and (+) construction corresponding to respectively untreated and treated samples with RNA 5' polyphosphatase. This enzyme specifically dephosphorylates 5′-triphosphorylated RNA such as primary RNA transcripts to convert them into 5′-monophosphorylated RNA (**e**). In the following RNA ligation step, the 3′ OH end of the RNA adapter will only ligate with 5′ monophosphate end RNA, which aims to enrich library (-) in TEX-resistant RNA compared to library (+) (**f**). Oligo(dT)-adapter primer was used to perform synthesis of first strand cDNA with M-MLV reverse transcriptase (**g**). Resulting cDNAs were PCR-amplified with high fidelity DNA polymerase and barcoded using 3′ sequencing adaptors (**h**). After cDNAs purification using the Agencourt AMPure XP kit (Beckman Coulter Genomics) (**i**) and quality-checked using capillary electrophoresis (Shimadzu MultiNA microchip electrophoresis system), the cDNA were pooled and sequenced on an Illumina NextSeq 500 system using 50 bp read length (**j**). As a result, reads are expected to mainly correspond to TEX-resistant processed RNA for library (-), and to enriched-primary RNAs (although TEX-resistant processed RNA are also present) for library (+). By aligned read count comparison between the two libraries, primary TSS will be identified from library (+) reads.

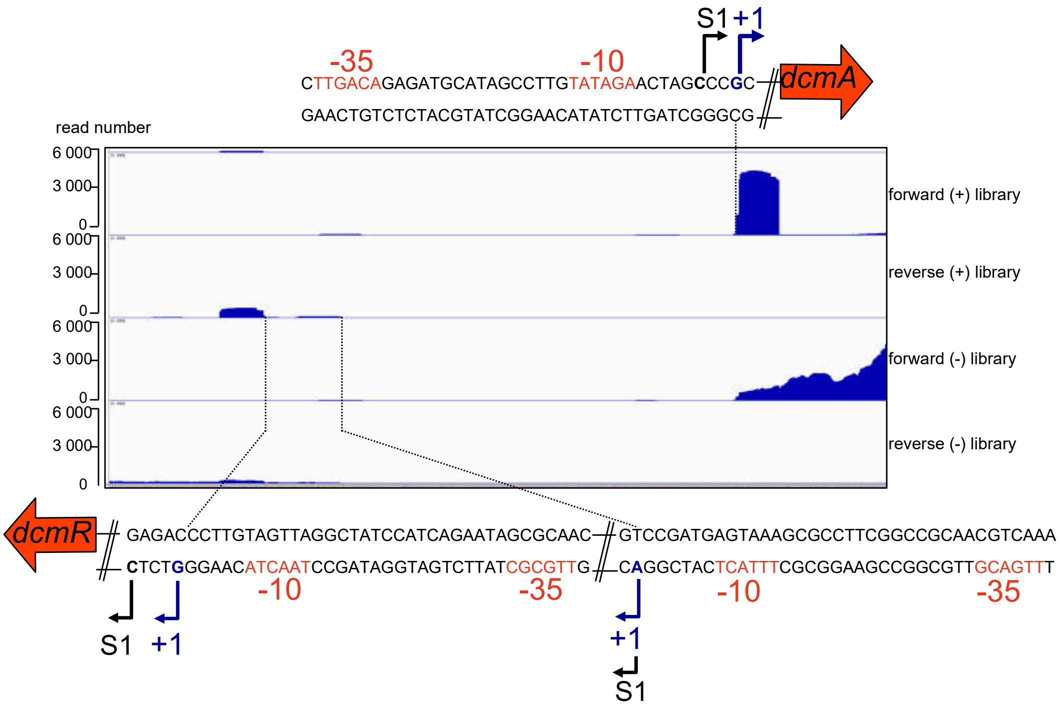

Figure S2. Start sites of the *dcmR-dcmA* intergenic region and comparison with previous data. Genes are divergently oriented. Nuclease S1 detected TSS (black arrows indicated as S1) [16] and dRNA-seq detected TSS (blue arrows indicated as +1). Graphical representation of the dRNA-seq read numbers are shown for each of the forward and reverse orientation of two libraries as explained in Figure S1.
